# Identification of the molecular mechanisms underlying brisket disease in Holstein heifers via microbiota and metabolome analyses

**DOI:** 10.1186/s13568-021-01246-0

**Published:** 2021-06-12

**Authors:** Kun Yao, Shuxiang Wang, Naren Gaowa, Shuai Huang, Shengli Li, Wei Shao

**Affiliations:** 1 College of Animal Science, Xinjiang Agricultural University, Urumqi, 830052 China; 2 Qinghai Academy of Animal Science and Veterinary Medicine , Qinghai University, Xining, 810016 China; 3 State Key Laboratory of Animal Nutrition, Beijing Engineering Technology Research Center of Raw Milk Quality and Safety Control, College of Animal Science and Technology, China Agricultural University, Beijing, 100193 China

**Keywords:** Brisket disease, Holstein heifer, High-altitude environment, Metabolomics, 16S rDNA sequencing

## Abstract

Brisket disease (BD) is common among Holstein heifers in high-altitude environments, and this disease may result in serious economic loss. At present, no effective treatment is available for brisket disease. In this study, liver and cecum samples were collected from five heifers with BD and five healthy heifers (HH) for analyses of the metabolome and microbiota. The mean pulmonary arterial pressure and systolic blood pressure were significantly higher in BD heifers, whereas the average breathing rate, blood oxygen saturation, and glucose level were significantly lower in BD group than in the HH group. Further, 16S rDNA data showed that the abundance of *Firmicutes* was significantly lower and that of *Bacteroidetes* was significantly higher in BD group than in the HH group. At the genus level, the BD group heifers harbored fewer *Ruminococcaceae* and *Lachnospiraceae* than the HH group. Several metabolites, including beta-d-fructose, d-ribose, 1,4-beta-d-glucan, sucrose, and glucose-6-phosphate were present at low levels in BD heifers. Moreover, the mean pulmonary arterial pressure was negatively correlated with beta-d-fructose (r =  − 0.74; *P* = 0.013), d-ribose (r =  − 0.72; *P* = 0.018), and acetyl-tyrosine-ethyl-ester (r =  − 0.71; *P* = 0.022). We also found that mean pulmonary arterial pressure was negatively correlated with most of the genera, including those in the families of Lachnospiraceae and Ruminococcaceae. In summary, the decreased levels of metabolites and microbial genera might affect BD by limiting the energy supply. This study may help us better understand the role of the microbiota in BD and provide new insights into the management of feeding to decrease the rate of BD in Holstein dairy cows in the Qinghai-Tibetan plateau.

**Supplementary Information:**

The online version contains supplementary material available at 10.1186/s13568-021-01246-0.

## Introduction

Yak, live in the Qinghai-Tibetan plateau, which is cold, has low air oxygen content, strong ultraviolet light, and a short forage-growing season. They provide the nomadic pastoralists with basic resources, including meat and milk. The extraordinarily harsh conditions in which these animals live results in low milk production and feed efficiency. This deficiency has resulted in milk shortages and necessitated the introduction of Holstein cows that produce milk with high efficiency (Qiao et al. 2013a, b). However, the high altitude and low atmospheric oxygen in this region have resulted in the development of brisket disease (BD) in Holstein heifers (Holt and Callan 2007; Malherbe et al. 2012). Approximately 3–5% of all heifers succumb to this disease. The mortality rate associated with BD is as high as 25% in high-altitude mountain ranges (Holt and Callan 2007; Malherbe et al. 2012). Presently, there are no effective measures to prevent or treat BD in Holstein cows. American dairy farmers mitigate BD-associated economic losses by monitoring pulmonary artery pressure and promptly selling off sick cows or transporting them to low altitudes for recovery (Williams et al. 2012)

BD is an idiopathic pulmonary edema that develops in Holstein cows that are poorly adapted to high-altitude hypoxic environments. It is associated with pulmonary hypertension, right heart failure, and death (Rhodes 2005). Cows raised at altitudes > 1500 m and presenting with an average pulmonary blood pressure of > 49 mmHg are diagnosed with high-altitude pulmonary hypertension (HAPH) (Holt and Callan 2007). The diagnostic criteria for BD include the clinical symptoms reported by Kuida et al. (1963), such as intrapleural, abdominal, pulmonary, renal, mesenteric, and recurring edema as well as hepatic congestion (Holt and Callan 2007). The pathogenesis of BD is characterized by HAPH caused by hypoxic pulmonary vasoconstriction and leading to irregular increases in pulmonary capillary pressure (Hopkins et al. 2005); pulmonary artery remodeling induced by hypoxia (Maggiorini and Leon-Velarde 2003); and hypoxia- and inflammation-mediated pulmonary vascular endothelial cell injury and changes in endothelial cell permeability (Densmore et al. 2006).

Intestinal microorganisms play vital roles in protecting the host health by preventing harmful substances from entering the blood and triggering systemic immune responses (Wan et al. 2019). In recent years, the role of the microbiota in the pathogenesis of pulmonary arterial hypertension (PAH) has been widely studied in depth. Thenappan et al. (2019) demonstrated that the early pathogenesis of PAH is correlated with the changes in the gut and circulating microbiota in the initiation of perivascular inflammation. In an animal study, animals with PAH showed an increased muscularis layer in the alimentary canal, increased intestinal permeability, decreased villus length, and fewer goblet cells, all of which were associated with changes in the associated microbial communities in the alimentary canals of the animals (Sharma et al. 2020). Sanada et al. (2020) identified some gut microbes that could suppress the development of PAH in rat models. Gaowa et al. (2020) identified potential microbial markers in the rumen of Holstein cows suffering from BD. However, the role of cecum microbiota in the pathogenesis of BD in Holstein cows has not yet been studied. Changes in the types and amounts of metabolites related to the alteration of the gut microbiota composition have been shown to impact the development of several diseases (Lu et al. 2014; Wang et al. 2011); these changes include those induced by the diet or environment. Given the role of the microbiota and its metabolites in the pathogenesis of diseases, we hypothesized that the gut microbiota-related metabolic changes would help elucidate the mechanism of BD in Holstein heifers.

We therefore used mass spectrometry-based metabolomic profiling to investigate the microbiota of heifers with BD and healthy heifers (HH) transferred from low-altitude to high-altitude environments. This study provides a theoretical basis for breeding BD-resistant Holstein heifers and developing strategies for BD prevention.

## Materials and methods

### Ethics statement

The Institution of Animal Care and Use Committee at the China Agricultural University (Beijing, China; Permit No. AW10102020-1-1) approved the protocol and methodology of the present study.

### Experimental design and sample collection

Overall, 2000 Holstein heifers aged 13–15 months were transported from a low altitude (1027 m; Xi’an, Shaanxi, China) to a high altitude (3658 m; Lhasa, Tibet, China). All the heifers were housed in the same barn and with free access to water, and were fed three times daily (07:00, 12:00, and 20:00) with a total mixed ration, that meet the maintenance and growth requirements of heifers according to the US National Research Council (2001) recommendations. The composition of the feed is listed in Additional file 4: Table S1. After 3 months of adaptation, 200 of the Holstein heifers began to manifest clinical symptoms of BD, including drooping ears, labored breathing, distended external jugular veins, and brisket and underjaw edema. Their standard pulmonary arterial pressure (PAP) was measured, and those with a good mental state, smooth breathing, PAP < 41 mmHg, and without any pronounced disease symptoms were considered healthy. Heifers manifesting BD exhibited all symptoms associated with the disease and had a PAP of ≥ 49 mmHg. Five heifers manifesting BD symptoms for 2 weeks were selected and included in the BD group, and five healthy heifers (HH) were chosen. All animals were aged 16–18 months and weighed 495 ± 15 kg, and none of them were pregnant.

Three hours after the morning feeding, the mean PAP (mmHg), diastolic blood pressure (mmHg), systolic blood pressure (mmHg), and heart rates (beats/min) were measured using an animal blood pressure monitor (Cardell Veterinary Monitor 9401BP; Sharn Veterinary Inc., Tampa, FL, USA). The tail was held securely to prevent it from swinging and the sphygmomanometer cuff was wrapped around the third condyle of the tail root. The airbag was then tightened to face the ventral side of the tail root. Measurements were taken after the cow had relaxed, repeated three times and the average of 20 pressure cycles was used (Gaowa et al. 2020). Pressure differences of < 10 mmHg were considered acceptable. Next, the vulva was opened and wiped, a sensor probe was inserted near the skin of the vulva, and blood oxygen saturation was measured using Nonin Avant 9600 (Nonin Medical Inc., Plymouth, MN, USA). Bidop ES-100V3 (Hadeco Inc., Kawasaki, Japan) was used to measure the blood flow velocity in the middle of the tail root vessel. After the cow had relaxed, two veterinarians observed its abdominal movements during breathing from the left and right sides of the tail. A single up-down motion was counted as one breath. All motions were counted and the average number of breaths per minute was calculated. Rectal temperatures were measured twice daily (07:15–11:00 and 14:15–18:15) using a digital thermometer (GLA M900; accuracy ± 0.1 °C; GLA Agricultural Electronics, San Luis Obispo, CA, USA). The thermometer was inserted into the rectum to a depth of ~ 10 cm and held in place for ~ 10 s until the temperature was recorded. Blood glucose levels were measured using a clinical automatic biochemical detector (7020; Hitachi, Chiyoda, Japan). Three consecutive measurements were taken and the average was recorded.

After all measurements were taken and recorded, the selected heifers were euthanized (Gaowa et al. 2020). Tissue samples were immediately collected from the liver, placed in liquid nitrogen and stored at − 80 ℃ until further analysis. The cecum contents were collected and stored at − 80 ℃ for 16S rDNA sequencing.

### 16S rDNA sequencing and sequencing data analysis

The DNA of all cecum content samples (1 g) was extracted using QIAamp DNA Stool Mini Kits (QS; Qiagen, Hilden, Germany) according to the manufacturer’s instructions. The extracted DNA was quantified using 1% agarose gel electrophoresis. The V3–V4 region of the bacterial 16S rDNA gene was amplified using the primers 338F (5′-ACTCCTACGGGAGGCAGCA-3′) and 806R (5′-GGACTACHVGGGTATCTAATC-3′). The PCR conditions were performed as follows: 95 °C for 3 min, followed by 27 cycles of denaturation at 95 °C for 30 s, annealing at 55 °C for 30 s, and extension at 72 °C for 45 s and a final extension at 72 °C for 10 min. The amplified products were detected using 1% agarose gel electrophoresis, and further purified via Agencourt AMPure XP beads (Beckman Coulter, Beverly, MA, USA). A library was constructed using a NEBNext Ultra DNA Sample Preparation Kit (NEB, Ipswich, MA, USA), the purified amplicons were sequenced on an Illumina MiSeq platform (Illumina, San Diego, CA, USA), and 250–300 bp paired-end reads were generated.

Short reads (< 230 bp), low-quality score (≤ 20) reads, and reads containing ambiguous bases, primer sequence mismatches, or barcode tags were removed, and the remaining reads were subjected to further analysis using QIIME (Caporaso et al. 2010). The reads were trimmed using the Illumina analysis pipeline (v. 2.6; Illumina) and separated using sample-specific barcode sequences. Operational taxonomic units (OTUs) were identified. The QIIME pipeline (Caporaso et al. 2011) was used for sequence analysis and α-diversity determination. Principal co-ordinate analysis (PCoA) and analysis of similarity (ANOSIM) were assessed using the Bray–Curtis distance algorithm and visualized using in the “vegan” package of R (https://cran.r-project.org/web/packages/vegan/). Biomarkers were detected using linear discriminant analysis (LDA) effect size (LEfSe) in Galaxy (https://huttenhower.sph.harvard.edu/galaxy). The thresholds were LDA > 3 and a *P-*value of < 0.05 was considered to indicate the identification of differences in genera. Kruskal–Wallis rank-sum test was used for differential genera identification. *P* < 0.05 is regarded as a different OTU or species.

### Metabolomic analysis based on ultrahigh-performance liquid chromatography–tandem quadrupole time-of-flight mass spectrometry

Liver samples weighing 50 mg were obtained from each of 10 heifers. The samples were treated with 1000 μL extraction solution (methanol:acetonitrile:water = 2:2:1 v/v/v) containing 2 μg/mL internal standard, homogenized thrice at 45 Hz for 4 min, subjected to ultrasound on ice for 5 min, and incubated at − 20 °C for 1 h to precipitate the proteins. The supernatants were then transferred to fresh Eppendorf tubes (Eppendorf, Hamburg, Germany) and centrifuged at 12,000 rpm at 4 °C for 15 min. The extracts were dried in a vacuum concentrator without heating and redissolved with 100 μL extraction solvent (acetonitrile:water = 1:1 v/v). The samples were vortexed for 30 s, sonicated in a 4 °C water bath for 10 min, and centrifuged at 12,000 rpm for 15 min at 4 °C. The supernatants were transferred to clean 2 mL liquid chromatography–mass spectrometry (LC/MS) glass vials for ultrahigh-performance liquid chromatography–tandem quadrupole time-of-flight mass spectrometry (UHPLC–QTOF-MS).

UHPLC–QTOF-MS was conducted using an Agilent 1290 Infinity LC UHPLC System (Agilent Technologies, Santa Clara, CA, USA) with an UPLC BEH Amide column (2.1 mm × 100 mm, 1.7 μm; Waters, Milford, MA, USA) coupled to a Triple TOF 6600 mass spectrometer (AB SCIEX, Foster City, CA, USA). The mobile phase consisted of 25 mM NH_4_Ac plus 25 mM NH_4_OH in water (pH 9.75) and acetonitrile. The experiments were performed using a previously reported gradient elution program (Zhang et al. 2020). Mass spectrometry was conducted in electrospray ionization-positive (ESI^+^) and negative (ESI^−^) modes using a dedicated ion source. Analyst TF (v. 1.7) (AB Sciex, Framingham, MA, USA) was used to continuously evaluate full-scan survey MS data. The ESI source conditions used were as previously described (Liu et al. 2020). ProteoWizard was used to convert the MS raw data into mzXML format. The data were then processed using the XCMS package of R v. 3.2 (R Core Team, Vienna, Austria) to determine the retention time, peak intensity, and mass-to-charge ratio (*m*/*z*). The CAMERA package in R v. 3.2 (R Core Team, Vienna, Austria) was used to annotate the peaks. An in-house MS2 database was used to identify the metabolites.

Data from the ESI^+^ and ESI^−^ modes were subjected to principal component analysis (PCA) with Pareto scaling. A partial least squares discrimination analysis (PLS-DA) was used to distinguish groups. PLS regression was used to establish associations between sample categories and metabolite expression for modeling and sample prediction (Szymańska et al. 2012). Variables important for the projection (VIP) were determined, to investigate the influence of metabolite expression patterns on sample classification and interpretation and screen for metabolic markers. Metabolites with VIP > 1, *P* < 0.05, and Log_2_|fold change|> 1 was considered to be significantly different. Kyoto Encyclopedia of Genes and Genomes (KEGG) analysis was performed on the differential metabolites.

### Correlation analysis among physiological parameters, metabolome, and 16S rDNA sequencing

Spearman correlation analysis was performed to determine the correlations between physiological parameters, different metabolites and genera using R software. We defined *P* < 0.05, |r|= 0.6–0.8 indicating significant relationship, and |r|> 0.8 as indicating extremely significant relationship.

### Statistical analysis

GraphPad Prism 8 (GraphPad Software, San Diego, CA, USA) and SPSS v. 22 (IBM, Armonk, NY, USA) were used for the statistical analyses. Data are presented as means ± standard deviations (SD). Pairwise group comparisons were conducted using independent-sample *t*-tests. A corrected *P* < 0.05 was considered to be significant.

## Results

### Physiological parameters

BD and HH Holstein heifers differed significantly in terms of mean PAP (*P* = 0.002), systolic blood pressure (*P* = 0.005), average breathing rate (*P* = 0.011), blood oxygen saturation (*P* = 0.034), and glucose level (*P* = 0.015) (Table 1). The mPAP and systolic blood pressure were significantly increased in BD. The levels of average breathing rate, blood oxygen saturation, and glucose levels were significantly decreased in BD. However, the heifers with and without BD did not differ significantly in terms of diastolic blood pressure (*P* = 0.170), heart rate (*P* = 0.760), or rectal temperature (*P* = 0.061).

**Table 1:** Physiological comparison in samples of BD and HH

Factors	HH (n = 5)	BD (n = 5)	*P*
mPAP (mmHg)	38.63 ± 1.56	74.73 ± 9.41	0.002
Systolic blood pressure (mmHg)	103.23 ± 1.57	120.17 ± 9.70	0.005
Diastolic blood pressure (mmHg)	52.65 ± 3.69	46.29 ± 8.61	0.170
Heart rate (beats/min)	90.56 ± 11.09	92.55 ± 8.80	0.760
Rectal temperature (°C)	38.68 ± 0.13	39.38 ± 0.56	0.061
Average breathing rate (breaths/min)	33.67 ± 6.08	17.20 ± 3.39	0.011
Blood oxygen saturation (%)	88.33 ± 4.13	76.40 ± 4.75	0.034
Glucose level (mmol/L)	3.91 ± 0.26	3.37 ± 0.24	0.015

Data exhibited as means ± standard deviation

*mPAP* mean pulmonary artery pressure, *BD* brisket disease, *HH* healthy heifer

*P* < 0.05 indicates significant difference

### Effect of brisket disease on cecum bacterial richness and diversity

A total of 1,761,336 raw reads were generated, ranging from 93,655 to 362,685 (Additional file 5: Table S2). The observed number of OTUs detected by the analysis reached 3,403 based on ≥ 97% sequence identity between reads, which was significantly higher in BD groups (*P* = 0.033; Table 2). The Good’s coverage estimates averaged more than 99%, implying that the current sequencing depth was sufficient to be representative of the microbiota studied (Table 2). When examining the community structure, we found that the richness of species observed (*P* = 0.033), and the Shannon (*P* = 0.002) and Simpson (*P* = 0.016) indices were significantly decreased in heifers with BD (Table 2), suggesting that the disease is connected to a reduction in microbial species richness and diversity.

**Table 2:** Alpha-diversity indices of BD and HH

Index	HH (n = 5)	BD (n = 5)	*P*
OTUs	2027 ± 153.84	1476.2 ± 399.38	0.033
Chao1	2484.01 ± 195.05	1887.44 ± 567.77	0.057
Goods coverage	0.99 ± 0.00	0.99 ± 0.00	0.347
Observed species	2026.20 ± 171.93	1476.16 ± 446.49	0.033
PD whole tree	131.49 ± 11.10	104.37 ± 28.73	0.084
Shannon	8.47 ± 0.29	6.83 ± 0.75	0.002
Simpson	0.99 ± 0.00	0.96 ± 0.02	0.016

*BD* brisket disease, *HH* healthy heifer, *PD* phylogenetic diversity

**Fig. 1:**
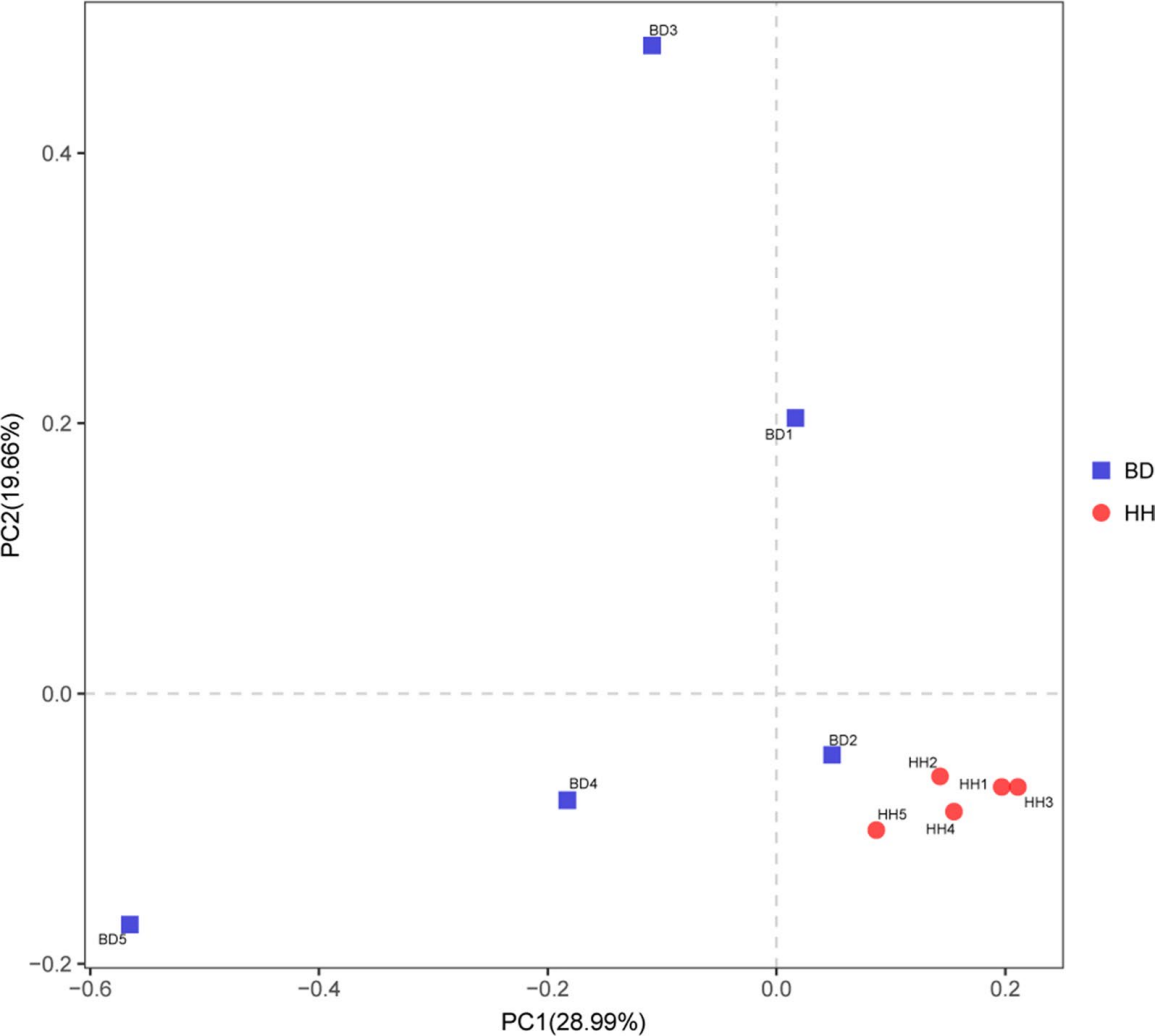
Comparison of the cecum microbiota by PCoA using the Bray–Curtis similarity index. *PCoA* principal co-ordinates analysis

Comparison of the cecum microbiota in the two groups was conducted using PCoA with the Bray–Curtis distance, and revealed a clear separation between the BD and HH groups (Fig. 1). To further investigate this phenomenon, we used ANOSIM. ANOSIM revealed a significant difference between BD and HH groups (R^2^ = 0.284; *P* = 0.011).

### Effect of brisket disease on cecum bacterial composition

We next compared the effects of BD on bacterial composition. We found that the *Firmicutes* and *Bacteroidetes* were the dominant phyla in the cecum, accounting for 94.18% of the total sequences (Table 3). A significantly lower relative abundance of *Firmicutes* (64.75% vs. 49.94%; *P* < 0.001) and higher relative abundance of *Bacteroidetes* (30.38% vs. 43.30%; *P* = 0.010) was observed in BD than in HH. At the genus level, *Ruminococcaceae*_*UCG*-005 was the dominant genus and was significantly decreased in BD (14.21% vs. 7.55%; *P* = 0.017). *Ruminococcaceae_UCG*-013 was also less abundant in BD than in HH (2.85% vs. 0.91%; *P* = 0.002).

**Table 3:** Taxonomic analysis at the phylum, family, and genus levels

Category	HH (n = 5)	BD (n = 5)	*P*
Phylum			
*Firmicutes*	64.75 ± 2.87	49.94 ± 7.31	< 0.001
*Bacteroidetes*	30.38 ± 5.49	43.30 ± 8.70	0.010
F/B	2.22 ± 0.62	1.23 ± 0.47	0.022
Family			
Ruminococcaceae	34.34 ± 4.94	17.84 ± 3.49	< 0.001
Rikenellaceae	15.69 ± 0.05	6.76 ± 4.29	0.006
Genus			
*Ruminococcaceae_UCG*-005	14.21 ± 5.32	7.55 ± 2.55	0.017
*Ruminococcaceae_UCG*-013	2.85 ± 0.97	0.91 ± 0.66	0.002
*Ruminococcaceae_UCG*-009	0.78 ± 0.130	0.37 ± 0.091	0.019
*Ruminococcus-2*	0.65 ± 0.088	0.17 ± 0.053	< 0.001
*Ruminococcaceae_UCG*-014	0.52 ± 0.150	0.07 ± 0.028	0.008
*Dorea*	0.33 ± 0.041	0.09 ± 0.022	< 0.001
*Marvinbryantia*	0.28 ± 0.061	0.07 ± 0.018	0.006
*Eubacterium_hallii_group*	0.26 ± 0.088	0.08 ± 0.018	0.043
*Blautia*	0.24 ± 0.078	0.06 ± 0.016	0.030
*Eubacterium_brachy_group*	0.22 ± 0.021	0.09 ± 0.032	0.004
*Lachnospiraceae_UCG*-001	0.13 ± 0.045	0.0212 ± 0.011	0.019
*Oscillospira*	0.11 ± 0.017	0.0470 ± 0.018	0.017
*Anaerosporobacter*	0.10 ± 0.037	5.60E−03 ± 3.100E−03	0.013
*Lachnospiraceae_FE2018*_group	0.04 ± 0.0114	9.70E−03 ± 4.000E−03	0.019
*Candidatus_Hepatincola*	0.02 ± 0.0110	3.48E−04 ± 3.000E−04	0.007
*Coprobacillus*	0.02 ± 4.60E−03	4.20E−03 ± 3.800E−03	0.017

We only list some of the genera

*BD* brisket disease, *HH* healthy heifer, *F/B* Firmicutes/Bacteroidetes

Significant differences were observed between BD and HH in terms of community composition. The cladogram in Additional file 6: Figure S1A shows differences in 13 taxa between BD and HH. The relative abundance of the classes *Gammaproteobacteria* and *Bacteroidia*, order *Bacteroidales*, family *Erythrobacteraceae*, and genera *Escherichia Shigella* and *Apibacter* were greater in BD than those in HH (Additional file 6: Figure S1B). In contrast, *Clostridiales*, Clostridia, *Ruminococcaceae*, *Firmicutes*, *Rikenellaceae*, and *Ruminococcaceae_UCG*-005 were significantly more abundant in HH than in BD.

### Metabolome analysis

**Fig. 2:**
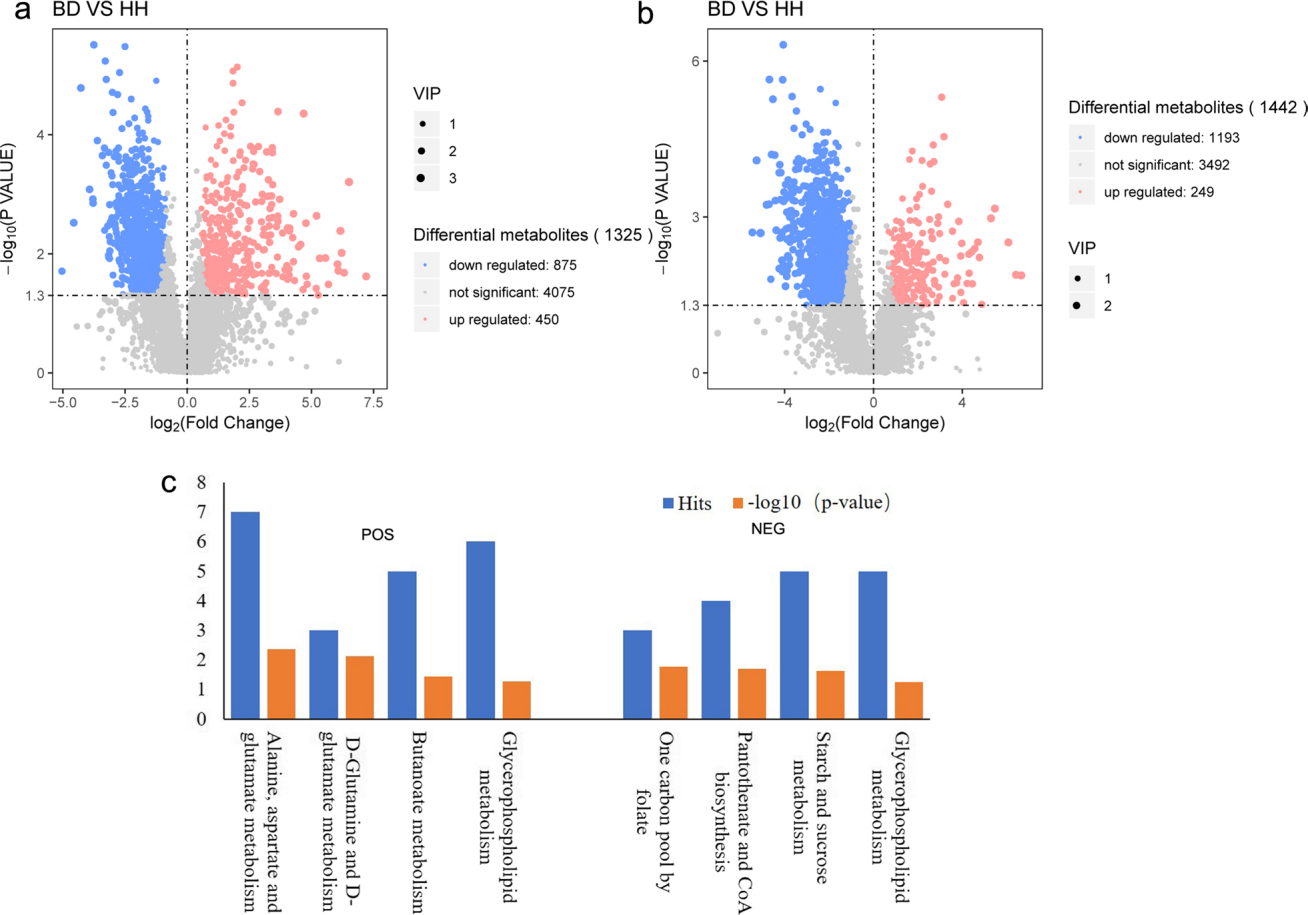
Scatter plots and KEGG analysis depicting metabolites identified in BD and HH. **a** Scatter plot of metabolites identified in ESI^+^ mode. **b** Scatter plot of metabolites identified in ESI^−^ mode. **c** KEGG analysis of different metabolites in the ESI^+^ and ESI^−^ modes. *KEGG* Kyoto Encyclopedia of Genes and Genomes, *BD* brisket disease, *HH* healthy heifer, *ESI*^*+*^ electrospray ionization-positive, *ESI*^*−*^ electrospray ionization-negative

We compared the hepatic metabolomic profiles of BD and HH using UHPLC–QTOF-MS. PCA and PLS-DA score plots revealed a clear separation between BD and HH (Additional file 7: Figure S2). In ESI^+^ mode, 1325 metabolites were significantly different in abundance between BD and HH. Of these, 450 were upregulated and 875 were downregulated in BD. In ESI^−^ mode, 1442 metabolites exhibited significantly different abundances between BD and HH. Of these, 249 were upregulated and 1,193 were downregulated in BD. Scatter plots depicting the differentially abundant metabolites are shown in Fig. 2A, B. All of the differentially abundant metabolites identified in ESI^+^ and ESI^−^ modes are listed in Additional files 1 and 2.

The differential metabolites detected in ESI^+^ mode were enriched mainly in d-glutamine and glutamate metabolism, alanine, aspartate and glutamate metabolism, butanoate metabolism, and glycerophospholipid metabolism pathways (*P* < 0.05; Fig. 2C). In ESI^−^ mode, the differential metabolites were enriched mainly in one carbon pool by folate, pantothenate and CoA biosynthesis, starch and sucrose metabolism, and glycerophospholipid metabolism.

Some of the carbohydrates as beta-d-fructose, d-ribose, 1,4-beta-d-glucan, sucrose, glucose-6-phosphate were significantly decreased in BD compared with HH. Amino acid and lipids, including l-asparagine, Ala-Gly, His-Ile, Glycyl-l-leucine, l-cysteine, and acetyl-tyrosine-ethyl-ester were also significantly decreased in BD compared with HH. Lys-Phe and l-leucyl-l-proline were increased in BD. We list some of the metabolites related to sugar, lipids, and amino acids in Table 4.

**Table 4:** Differential metabolites were identified in BD by mass spectrometry

MS2.name	VIP	*P*-value	Log_2_(fold change)
Beta-d-fructose	1.92	< 0.001	− 2.89
d-ribose	2.25	0.015	− 1.14
1,4-beta-d_glucan	1.18	0.019	− 1.20
Sucrose	1.12	0.002	− 1.09
Glucose-6-phosphate	1.25	0.014	− 1.25
l-Asparagine	1.03	0.010	− 0.83
Ala-Gly	2.13	0.012	− 2.90
His-Ile	2.00	0.004	− 2.65
Lys-Phe	2.05	0.017	3.62
l-leucyl-l-proline	2.28	0.004	4.07
Glycyl-l-leucine	1.99	< 0.001	− 3.29
l-Cysteine	1.12	0.033	1.09

*VIP* variable important for the projection, *MS* mass spectrometry, *BD* brisket disease, *HH* healthy heifer

### Correlation analysis among physiological parameters, metabolome, and microbiota

**Fig. 3:**
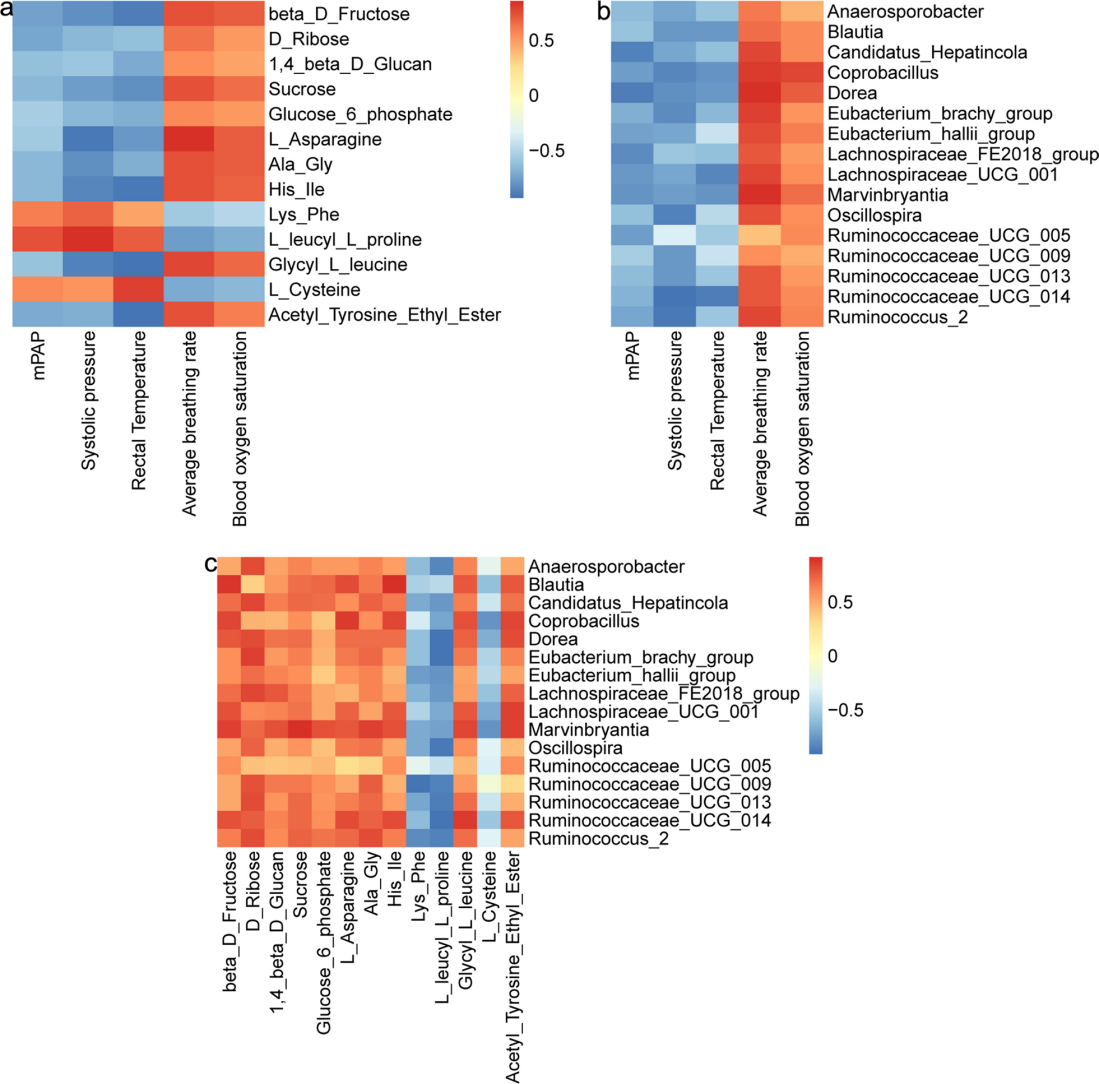
Analysis of correlations among physiological parameters, metabolites, and genera. **a** The correlation analysis between physiological parameters and metabolites. **b** The correlation analysis between physiological parameters and genera. **c** The correlation analysis between genera and metabolites. Red color intensity increases as correlation approach 1. Blue color intensity increases as correlation approach − 1. We defined |r|= 0.6–0.8 as significantly related and |r|> 0.8 as extremely significantly related

We can observe from the Fig. 3A that mPAP is negatively correlated with beta-d-fructose (r = − 0.74; *P* = 0.013), d-ribose (r = − 0.72; *P* = 0.018), 1,4-beta-d-glucan (r = − 0.62; *P* = 0.053), sucrose (r = − 0.66; *P* = 0.037), glucose-6-phosphate (r = − 0.55; *P* = 0.098), l-Asparagine (r = − 0.56;* P* = 0.090), Ala-Gly (r = − 0.66; *P* = 0.037), His-Ile (r = − 0.64; *P* = 0.042), Glycyl-l-leucine (r = − 0.60; *P* = 0.067), and acetyl-tyrosine-ehyl-ester (r = − 0.71; *P* = 0.022). Moreover, it has positive correlation with Lys-Phe (r = 0.59;* P* = 0.074), l-leucyl-l-proline (r = 0.74; *P* = 0.013), and l-Cysteine (r = 0.55; *P* = 0.098). Systolic pressure and rectal temperature have similar correlation partner with mPAP. Moreover, average breathing rate and blood oxygen saturation have opposed correlation partner with mPAP. We also found that PAP was negatively correlated with most of the genera, including *Candidatus_Hepatincola* (r = − 0.85; *P* = 0.002), *Coprobacillus* (r = − 0.73; *P* = 0.016), Dorea (r = − 0.86; *P* = 0.001), *Eubacterium*_hallii_group (r = − 0.72; *P* = 0.018), *Lachnospiraceae*_FE2018_group (r = − 0.82; *P* = 0.004), *Lachnospiraceae_UCG*-001 (r = − 0.76; *P* = 0.011), *Marvinbryantia* (r = − 0.78;* P* = 0.007), *Ruminococcaceae_UCG*-005 (r = − 0.74; *P* = 0.013), *Ruminococcus*_2 (r = − 0.70; *P* = 0.025) (Fig. 3B). Those genera was positively correlated with beta-d-fructose, d-ribose, 1,4-beta-d-glucan, sucrose, glucose-6-phosphate, l-Asparagine, Ala-Gly, Glycyl-l*-*leucine, and acetyl-tyrosine-ethyl-ester, and l-leucyl-l-proline (Fig. 3C). The detailed r values of the correlation analyses are list in Additional file 3.

## Discussion

**Fig. 4:**
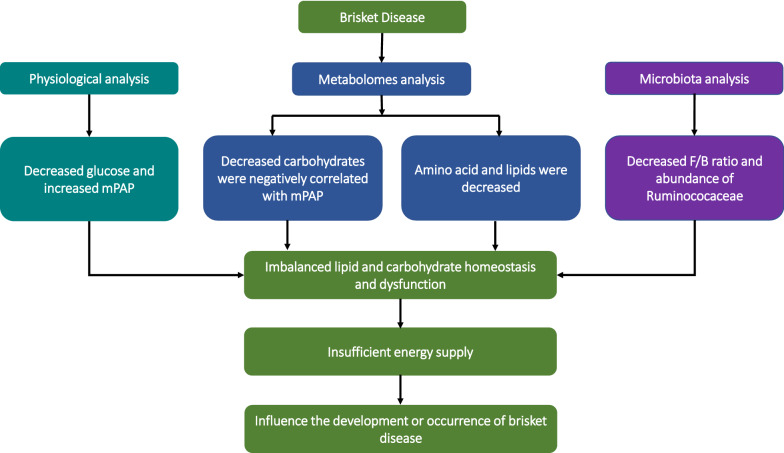
The hypothesis mechanism of occurrence or development of BD. *MPAP* mean pulmonary artery pressure, *F/B* Firmicutes/Bacteroidetes

This study is, to the best of our knowledge, the first to investigate the details of the pathogenesis in Holstein cows using insights from the metabolome and microbiota. We found that imbalances in lipid and carbohydrate, homeostasis, and subsequent dysfunction of these systems, contributed significantly to the occurrence and development of BD (Fig. 4).

The metabolites of Holstein cows suffering from HAPH were significantly enriched in the KEGG pathway terms associated with metabolism and biosynthesis. Fatty acid oxidation and malonyl CoA decarboxylase play critical roles in the metabolic processes involved in pulmonary hypertension. Thus, they are potential targets for the development of therapeutic agents against HAPH (Sutendra et al. 2010). N-3 fatty acids are associated with significant increases in HAPH-associated mortality. They inhibit hepatic lipogenesis, and increase blood nitric oxide levels (Rostami et al. 2016). Nitric oxide maintains the pulmonary vasculature and decreases pulmonary vascular resistance (Fagan et al. 2001; Tonelli et al. 2013). Therefore, the decreased levels of metabolites in the BD group were primarily related to pulmonary metabolism and, might therefore play important roles in the adaption of animals to at high altitudes.

Metabolites involved in starch and sucrose metabolism were significantly downregulated in the BD group compared with the HH group, and the Holstein cows with BD had relatively low carbohydrate levels. Some carbohydrates, lipids and amino acids were significantly decreased in the BD group compared with HH. These energy suppliers were negatively correlated with mPAP, indicating that they play roles in BD. A previous study demonstrated that NADPH redox and glucose-6-phosphate dehydrogenase are involved in hypoxic pulmonary vasoconstriction. These molecules may also be responsible for increases in PAP levels associated with the development of pulmonary hypertension (Gupte et al. 2010). Plasma galactose levels were lower in BD than in HH heifers, an observation which indicates that hypoxia might alter glucose metabolism. Lactate and glucose are two major sources of energy in most organisms. The metabolic profiles observed in this study were consistent with the inhibition of aerobic glucose oxidation, and their levels increased during hypoxia. However, glycogenolysis and gluconeogenesis were also enhanced under hypoxic conditions (Zhu et al. 2015). The levels of l-Asparagine, Ala-Gly, His-Ile, Glycyl-l-leucine, and acetyl-tyrosine-ethyl-ester were significantly lower in BD than those in HH heifers, and negatively correlated with mPAP, indicating that compounds may prevent energy production at high altitudes, ultimately resulting in energy insufficiency and causing BD. This result was consistent with that of a previous study into humans manifesting acute mountain sickness (Zhu et al. 2015). These findings indicate that decreased levels of glucose and lipid metabolites lowered energy production in heifers at high altitudes and may have contributed to the development and progression of BD.

Tissue function and overall health are affected by metabolic changes that occur in the intestinal microbiota in response to nutritional interventions (Putignani et al. 2016). Intestinal microbiota has also been shown to in the adaptation of organisms to high altitudes (Lan et al. 2017; Li and Zhao 2015; Quagliariello et al. 2019; Zhang et al. 2018a). Bacteroidetes and Firmicutes were the predominant phyla in the cecum in the present study, finding in agreement with previous studies on rumen and feces (Gaowa et al. 2020; Zhang et al. 2018b). Previous studies reported that the Firmicutes/Bacteroidetes (F/B) ratio was involved in energy harvesting and body fat storage in humans and mice (Ley et al. 2006; Turnbaugh et al. 2006). Turnbaugh et al. (2006) found that obese mice with higher F/B ratios showed strong effect on harvesting energy. Therefore, we assume that BD heifers with lower F/B ratios might have obstructions to the harvesting or utilization of energy. The relative abundance of *Bacteroidetes* was significantly elevated in the intestine of BD heifers, and was positively associated with insulin resistance. Johnson et al. (2017) demonstrated that the body’s response to glucose increased with an average in the abundance of Bacteroidetes. Enhanced insulin resistance could result in lower insulin efficiency, and inhibit glucose uptake and utilization. Combined with the metabolome analysis, we concluded that decreased F/B ratio and glucose metabolism suppressed the harvesting and utilization of energy, potentially leading to the development of BD.

Differences in the relative abundances of the members of the microbial community are attributed mainly to diet, followed by host and geographical environment (Henderson et al. 2015). However, the health status of the host might also be a major determinant of the structure of the microbial community. In the present study, substantial differences were observed between BD and HH in terms of microbial community richness and diversity despite the fact that all animals were provided with the same diet, and were exposed to the same geographical environment. LEfSe analysis indicated that *Escherichia Shigella* and *Apibacter* were present in greater abundance in BD than in HH, and might therefore be useful biomarkers of the condition. Lachnospiraceae and Ruminococcaceae are the most abundant Firmicute families in the gut environment,and Lachnospiraceae was reported to be depleted in inflammatory bowel disease (Biddle et al. 2013), an observation which might indicate that *Lachnospiraceae* is negatively connected with inflammatory. *Lachnoclostridium* and *Ruminococcaceae* have also been reported to be strongly linked to the regulation of the immune system (Lee et al. 2020). In the present study, the significant decrease in the abundance of the *Lachnospiraceae*_FE2018_group and *Lachnospiraceae_UCG*-001 in BD might also imply a role for *Lachnospiraceae* in BD via an increase in inflammation. *Ruminococcus* is a major contributor of carbohydrate-active enzymes (Rosewarne et al. 2012) that break down plant cell walls and degrade dietary cellulose, and pectin (Kala et al. 2017; Lim et al. 2013). Rats exposed to hypobaric and hypoxic conditions have been found to be deficient in intestinal *Ruminococcus* (Xu et al. 2014). In this study, we found that the amount of *Rumenococcus* was significantly reduced in Holstein cows with BD, and that *Rumenococcus* had a positive association with the presence of carbohydrates, and a negative correlation with pulmonary hypertension. These observations further confirmed the role of carbohydrates as energy supplies in BD. A combination of quercetin and resveratrol has been found to could alleviate diet-induced inflammation and obesity, partly by increasing the level of *Ruminococcaceae_UCG*-005 and *Ruminococcaceae_UCG*-014 (Zhao et al. 2017). *Ruminococcaceae_UCG*-005 dominated the gut microbiome, and significantly decreased BD might play an essential role in BD. Therefore, the decreased abundance of *Ruminococcaceae_UCG*-005 and *Ruminococcaceae_UCG*-014 in BD might be related to inflammation. In combination, *Lachnospiraceae* and *Ruminococcaceae* may influence BD by enhancing inflammation and decreasing the energy supply by regulating metabolites.

In conclusion, our findings indicate that decreases in specific metabolites and microbial genera in the gut regulated glucose and lipid metabolism and lowered energy production in heifers living at high altitudes. These differences in the metabolome and microbiome may have contributed to the development and progression of BD.

## Supplementary Information

**Additional file 1**: The list of all differential metabolites identified in positive ion mode.

**Additional file 2**: The list of all differential metabolites identified in negative ion mode.

**Additional file 3**. The detailed r values of correlation analyses.

**Additional file 4: Table S1**. Ingredient and nutrient concentrations of experimental diets.

**Additional file 5: Table S2**. The raw data of fecal samples of BD and HH groups. BD, brisket disease. HH, healthy heifers.

**Additional file 6: Figure S1.**. LEfSe based on classification information and LDA scores distribution histogram. (A) LEfSe of BD and HH. Circle radiating from inside to outside represents classification level from phylum to genus. (B) LDA score distribution diagram of BD and HH. Histogram length represents the impact of significantly different species. Red node and bar indicate microbial group playing important role in BD. Green node and bar indicate microbial group playing important role in HH. LDA, linear discriminant analysis; LEfSe, LDA effect size.

**Additional file 7: Figure S2.**. The PCA and PLS-DA score plots at positive and negative mode in BD and HH groups. (A) the PCA analysis at positive mode in BD and HH groups. (B) the PLS-DA analysis at positive mode in BD and HH groups. (C) the PCA analysis at negative mode in BD and HH groups. (D) the PLS-DA analysis at negative mode in BD and HH groups. PCA, principal component analysis. PLS-DA, partial least squares discrimination analysis. BD, brisket disease; HH, healthy heifers.

## Data Availability

The sequence data for the 16S rDNA sequencing was submitted to the NCBI-SRA database with the accession numbers of PRJNA673101.

## Abbreviations

BD: Brisket disease
HH: Healthy heifers
mPAP: Mean pulmonary arterial pressure
HAPH: High-altitude pulmonary hypertension
PAH: Pulmonary arterial hypertension
PCoA: Principal co-ordinates analysis
OTU: Operational taxonomic units
ANOSIM: Analysis of similarity
LDA: Linear discriminant analysis
LEfSe: LDA effect size
UHPLC–QTOF-MS: Metabolomic analysis based on ultrahigh-performance liquid chromatography–tandem quadrupole time-of-flight mass spectrometry
ESI^+^: Electrospray ionization-positive
ESI^−^: Electrospray ionization-negative
PCA: Principal component analysis
PLS-DA: Partial least squares discrimination analysis
VIP: Variables important for the projection
SD: Standard deviation
KEGG: Kyoto Encyclopedia of Genes and Genomes
