# Supplementary material for: Identification of the molecular mechanisms underlying brisket disease in Holstein heifers via microbiota and metabolome analyses
Source: AMB Express. 2021 Jun 12;11:86. doi: 10.1186/s13568-021-01246-0 (PMC8241945; doi:10.1186/s13568-021-01246-0)
Supplement: Supplementary file 4 — Additional file 4: Table S1. Ingredient and nutrient concentrations of experimental diets. [file 13568_2021_1246_MOESM4_ESM.docx]

Table S1. Ingredient and nutrient concentrations of experimental diets.

| **Items** | **Content** |
| --- | --- |
| Ingredient/diet (g/100g DM) | |
| Corn silage | 36.40 |
| Alfalfa hay | 24.36 |
| Oat hay | 9.56 |
| Corn grain | 14.91 |
| Soybean meal | 4.47 |
| Rapeseed meal | 2.09 |
| Cotton seed meal | 1.79 |
| Wheat bran | 1.82 |
| Cotton seed protein | 1.49 |
| Jujube powder | 1.04 |
| Premix | 0.30 |
| Limestone | 0.50 |
| Dicalcium phosphate | 0.42 |
| Sodium bicarbonate | 0.75 |
| Salt | 0.24 |
| Chemical composition (g/100g DM) | |
| Crude protein | 13.49 |
| NE_L_ (MJ/kg) | 6.02 |
| Neutral detergent fiber | 34.11 |
| Acid detergent fiber | 22.96 |
| Ether extract | 3.50 |
| Ash | 6.40 |
| Calcium | 0.73 |
| Total phosphorus | 0.47 |

Note: Every kilogram of premix contained: vitamin A, 1,000,000 IU; Vitamin D, 65,000 IU; Vitamin E, 5,000 IU; Fe, 2,000 mg; Mn, 2,550 mg; Zn, 5,500mg; Cu, 1,750mg; Co, 40mg; I, 70mg; and Se, 75 mg.
