# Supplementary material for: Identification of the molecular mechanisms underlying brisket disease in Holstein heifers via microbiota and metabolome analyses
Source: AMB Express. 2021 Jun 12;11:86. doi: 10.1186/s13568-021-01246-0 (PMC8241945; doi:10.1186/s13568-021-01246-0)
Supplement: Supplementary file 5 — Additional file 5: Table S2. The raw data of fecal samples of BD and HH groups. BD, brisket disease. HH, healthy heifers. [file 13568_2021_1246_MOESM5_ESM.docx]

Table S2. The raw data of fecal samples of BD and HH groups.

| Sample ID | Raw tags | Clean tags |
| --- | --- | --- |
| BD1 | 159,128 | 148,583 |
| BD2 | 122,795 | 116,454 |
| BD3 | 180,375 | 163,519 |
| BD4 | 153,493 | 136,314 |
| BD5 | 156,383 | 138,580 |
| HH1 | 131,293 | 120,040 |
| HH2 | 213,133 | 198,902 |
| HH3 | 362,685 | 351,481 |
| HH4 | 188,396 | 176,250 |
| HH5 | 93,655 | 76,050 |

BD, brisket disease; HH, health heifers.
