# Supplementary figures and images for: Identification of the molecular mechanisms underlying brisket disease in Holstein heifers via microbiota and metabolome analyses
Source: AMB Express. 2021 Jun 12;11:86. doi: 10.1186/s13568-021-01246-0 (PMC8241945; doi:10.1186/s13568-021-01246-0)

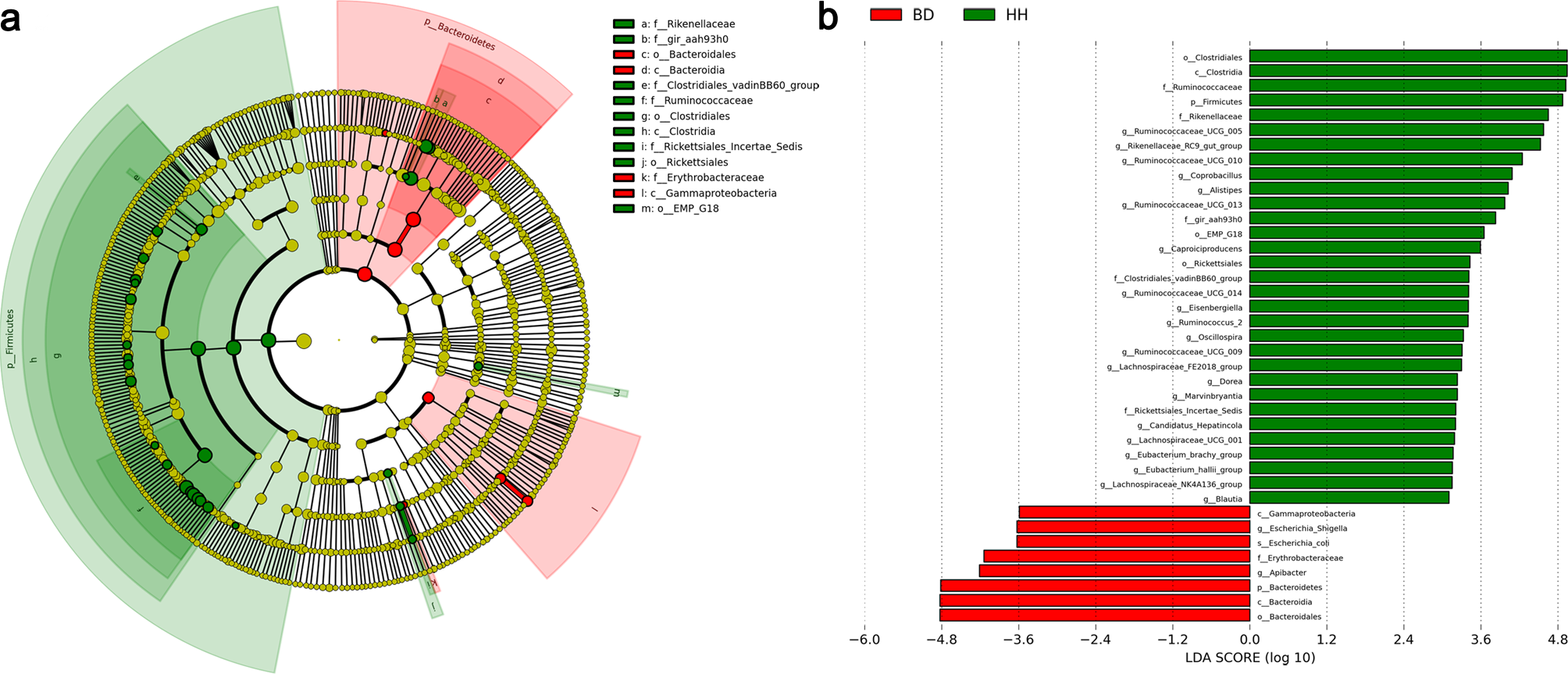

Supplement: Supplementary file 6 — Additional file 6: Figure S1.. LEfSe based on classification information and LDA scores distribution histogram. (A) LEfSe of BD and HH. Circle radiating from inside to outside represents classification level from phylum to genus. (B) LDA score distribution diagram of BD and HH. Histogram length represents the impact of significantly different species. Red node and bar indicate microbial group playing important role in BD. Green node and bar indicate microbial group playing important role in HH. LDA, linear discriminant analysis; LEfSe, LDA effect size. [file 13568_2021_1246_MOESM6_ESM.jpg]

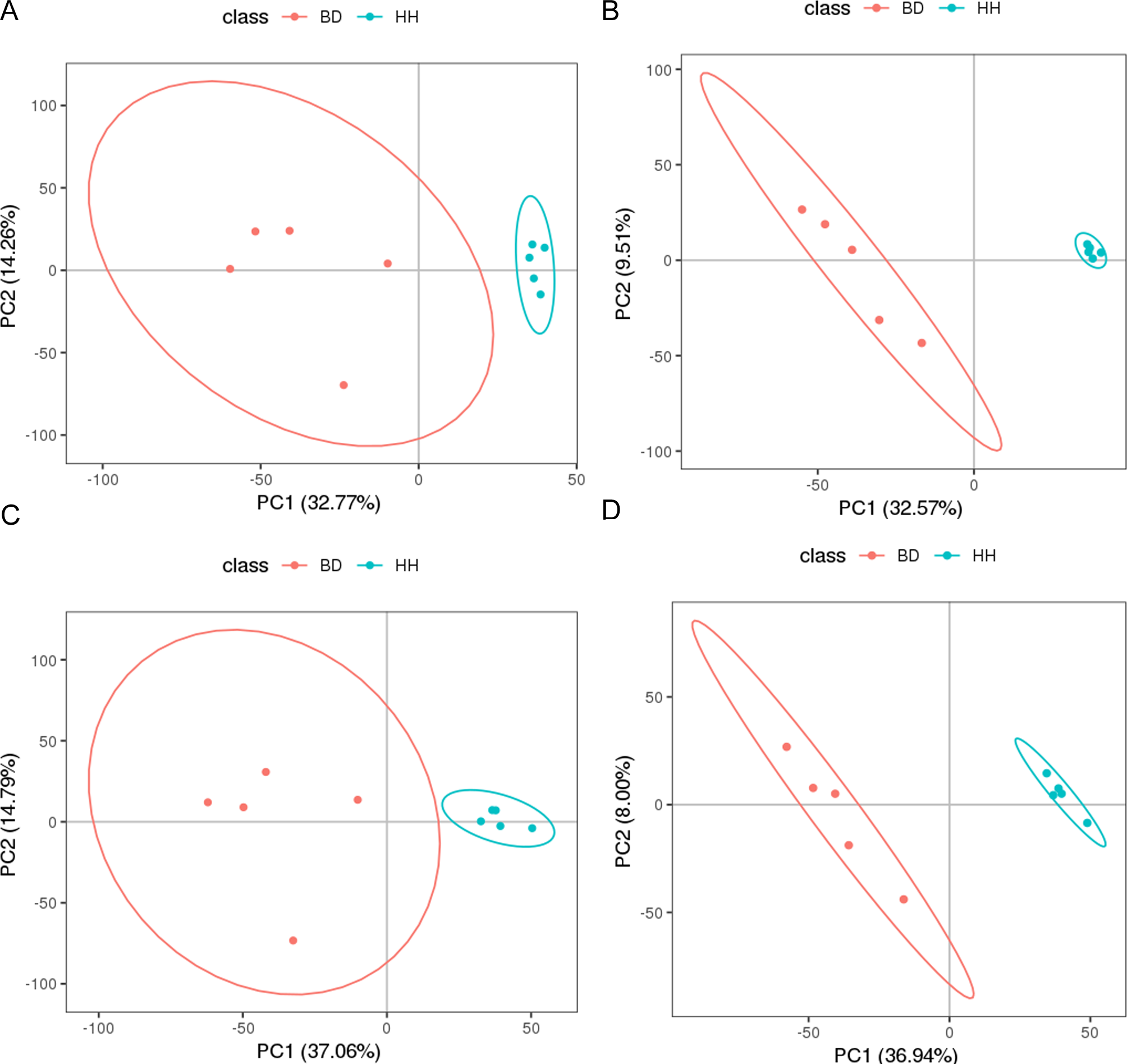

Supplement: Supplementary file 7 — Additional file 7: Figure S2.. The PCA and PLS-DA score plots at positive and negative mode in BD and HH groups. (A) the PCA analysis at positive mode in BD and HH groups. (B) the PLS-DA analysis at positive mode in BD and HH groups. (C) the PCA analysis at negative mode in BD and HH groups. (D) the PLS-DA analysis at negative mode in BD and HH groups. PCA, principal component analysis. PLS-DA, partial least squares discrimination analysis. BD, brisket disease; HH, healthy heifers. [file 13568_2021_1246_MOESM7_ESM.jpg]
